# What is the impact of rural bank credit access on the technical efficiency of smallholder cassava farmers in Ghana? An endogenous switching regression analysis

**DOI:** 10.1016/j.heliyon.2021.e07102

**Published:** 2021-05-21

**Authors:** Arnold Missiame, Rose A. Nyikal, Patrick Irungu

**Affiliations:** Department of Agricultural Economics, University of Nairobi, Kenya

**Keywords:** Technical efficiency, Credit access, Rural and community banks, Endogenous switching regression, Stochastic frontier model

## Abstract

This paper assesses the impact of access to credit from rural and community banks (RCBs) on the technical efficiency of smallholder cassava farmers in Ghana. The study employed the stochastic frontier, and endogenous switching regression models to estimate the technical efficiency, and the impact of RCB credit access, respectively, on a randomly selected sample of 300 smallholder cassava farmers in the Fanteakwa District of Ghana. Results suggest that cassava farmers in the District are 70.5 percent technically efficient implying that cassava yield levels could be increased further by 29.5 percent without changing the current levels of inputs. The results further reveal that the gender of the household head, access to extension services, membership in farmer organizations, and proximity to the bank are the major factors that positively influence farmers to access credit from RCBs. On average, farmers who accessed credit from RCBs have significantly higher technical efficiencies than farmers who did not access, suggesting that access to credit from RCBs positively impacts the technical efficiency of smallholder cassava farmers.

## 1 Introduction

The importance of cassava as a global food crop can be evidenced in its quantity produced globally. In 2013, approximately 276 million metric tonnes (MT) were recorded, out of which Africa accounted for approximately 158 million MT, representing about 57 percent of the world's cassava production in 2013 (Koyama et al., 2015).

In Ghana, the crop contributes 22 percent to the country's agricultural gross domestic product (AGDP). It is consumed in all the sixteen administrative regions of the country, thereby serving as an important food security crop (Ghartey et al., 2014). Between 2008 to 2010, the country experienced tremendous increases in cassava yield. These increases were attributed to the introduction of disease-resistant and high-yield varieties. However, current production levels stand at 20 million MT which is less than the potential yield of 28 million MT (FAOSTAT, 2018). This gap has been partially blamed on constraints such as diseases and pests, poor agronomic practices, and limited technical know-how. Furthermore, the agricultural sector of Ghana, particularly, the crop subsector is dominated by resource-poor smallholder farmers who are responsible for about 80 percent of total crop production in the country. They are faced with several constraints, including the persistent lack of or limited access to credit. Studies have shown that yields can likely be increased if improved inputs are used and better agronomic practices are adhered to (Koyama et al., 2015).

According to Koyama et al. (2015), any extra gains in yields would require an estimated additional cost of $200 per hectare for improved cassava stem cuttings, fertilizer, pesticides, and labor costs. That notwithstanding, access to credit continues to remain a major issue facing smallholder farmers. A study by Abor and Biekpe (2006), on small business financing schemes in Ghana, found that more than 50 percent of small firms (which also includes smallholder farmers) in Ghana were unaware of the financing schemes available to them. This raises questions about whether smallholder farmers in the country are aware of rural and community banks (RCBs) and their credit programs. The Government of Ghana established RCBs with the aim of providing financial services, particularly, credit to players in the agricultural value chain, who are largely located in rural areas.

Several studies have investigated the factors influencing smallholder farmers' demand for, and use of formal credit. Conclusions drawn from most of these studies are that smallholder farmers' socioeconomic, demographic, and farm-level characteristics influence their demand for, and use of formal credit (Ullah et al., 2020; Assogba et al., 2017; Buah et al., 2011; Baffoe and Matsuda, 2015; Akudugu, 2016). Other studies have also tried to evaluate how credit impacts the welfare of farmers (Lakhan et al., 2020; Amanullah et al., 2019). Further, there are several studies on the technical efficiency of farmers of crops such as maize, rice, and cocoa (Danso-Abbeam et al., 2020; Ali et al., 2019; Martey et al., 2019; Abdulaai et al., 2018; Anang et al., 2017). However, there is little or no empirical evidence of the technical efficiency of cassava farmers, and most importantly, the impact of access to credit from RCBs on the technical efficiency of rural smallholder cassava farmers in rural Ghana; considering that currently, there are about 140 RCBs with close to 1000 branches scattered all over the country. This leaves a gap in knowledge regarding smallholder farmers’ access to credit and their technical efficiencies.

Against this backdrop, this present study assesses the impact of access to credit from RCBs on the technical efficiency of smallholder cassava farmers in rural Ghana. The study, accordingly, has two specific objectives: (1). to estimate the technical efficiency of smallholder cassava farmers in rural Ghana, and; (2) to measure the impact access to RCB credit has on the technical efficiency of the smallholder cassava farmers in rural Ghana. This study is an attempt to provide new information and bridge the gap in knowledge on rural credit access and its link with technical efficiency; and at the same time provide evidence that will aid policymakers across the sub-region, in formulating policies specific to credit access and enhancements in the efficiency of cassava farmers.

The rest of the paper is organized as follows: Section 2 presents the methodology consisting of an overview of the stochastic frontier and the endogenous switching regression models, empirical models, and the data used; Section 3 presents a discussion of the results. The paper ends with conclusions and policy implications of the findings.

## 2 Literature review

Access to credit may not directly impact productivity. However, it may indirectly impact productivity through the positive effects on smallholder farmers’ adoption of agricultural technologies (Mariano et al., 2012). It may also impact productivity, indirectly, through hired skilled labor, improved health care, and increased capital for farm investments. Jimi et al. (2019) elucidate how crucial access to credit is for smallholder farmers by stating that it aids the farmers in the adoption of improved technology.

Empirically, the relationship between agricultural credit access and technical efficiency has been studied extensively through different approaches with different underlying assumptions. For instance, in Pakistan, Chandio et al. (2017) investigated the impact of agricultural credit and farm size on rice productivity using the stochastic frontier approach. The study found that credit played a dominant role in enhancing the technical efficiency of rice farmers. Also, in Pakistan, Akram et al. (2013) employed the stochastic frontier model to evaluate the effect of agricultural credit on the production efficiency of smallholder farmers. The study found that the mean technical efficiency was 0.90 for credit users and 0.79 for non-users. The high technical efficiency of credit users was due to their timely access to farm inputs. Ullah et al. (2019) assessed the technical efficiency of open shed broiler farms in Pakistan using the stochastic frontier model. The study reported that credit, among other factors, has a significantly negative effect on the technical inefficiency of the farms. In Vietnam, Long et al. (2020) employed the double bootstrap data envelopment analysis (DEA) to assess the cost and the technical efficiency of aquaculture. The study found that credit constraints negatively affect both cost and technical efficiency.

Zakaria et al. (2019) employed the Cobb-Douglas production function and panel data to investigate the impact of financial development on agricultural productivity in South Asia. The study treated financial development as one of the inputs in the Cobb-Douglas production function. The results revealed an inverted U-shaped relationship between financial development and agricultural productivity. It suggests that agricultural productivity increases initially and then falls eventually with improvements in financial development. In China, Jianmei and Barry (2014) employed the bootstrapped DEA approach to investigate the effect of formal credit access on technical efficiency. The study found that demand-side credit constraints have detrimental impacts on household technical efficiency. Lakhan et al. (2020) utilized the treatment effect model to measure the impact of credit constraints on the welfare of wheat farmers in agrarian economy. Using a random sample of 575 wheat farmers from the Sindh province of Pakistan, the study reported that the income of credit-constrained farmers is about 13.8 percent lower than that of unconstrained farmers.

In the Sub-region, Awotide et al. (2015) investigated how credit access impacts agricultural productivity in Nigeria. Using the endogenous switching regression (ESR) model to analyze household data from a sample of 841 smallholder cassava farmers, the study found that access to credit positively enhanced cassava productivity. Bokpin et al. (2018) explored the effect of financial access on firm productivity in SSA, using the semi-parametric approach. The study revealed that firms’ access to cost-effective credit facilities affects their productivity positively.

In Ghana, Abate et al. (2016) used the propensity score matching technique (PSM) to evaluate the impact of rural finance on smallholder farmers' adoption of improved agricultural technologies in Ethiopia. The study found that access to institutional financial services positively impacts agricultural technology adoption. Martey et al. (2019) also employed the PSM to measure the impact of credit on the technical efficiency of 223 smallholder maize farmers in the Northern part of Ghana. The study revealed that credit positively impacted the technical efficiency of the farmers. Nkegbe (2018) employed the two-stage double bootstrap DEA method to determine the relationship between credit access and smallholder crop farmers’ technical efficiency. From a sample of 445 households, the study found that farmers could achieve 50 percent of their potential output due to credit access. Akudugu (2016) also investigated the effect of access to credit on the agricultural productivity of households, using a hierarchical competitive model. The findings revealed that household agricultural productivity is influenced positively by access to credit.

We observe that, the studies reviewed employed either one of two approaches. On the one hand, is the stochastic frontier or the DEA methods. In the stochastic frontier approach, access to credit is treated as a covariate in the inefficiency model. In the DEA method, access to credit is observed as one of the factors of production. On the other hand, are studies that employed the PSM technique. Although this method allows a comparison between farmers who accessed credit and farmers who did not access it, the approach faces the issue of selection bias (Coulibaly et al., 2017; Asfaw et al., 2012; Shiferaw et al., 2014; Khonje et al., 2015). This current study employs the ESR model to investigate the impact of access to credit, particularly, from RCBs on the technical efficiency of cassava farmers in Ghana.

## 3 Methods

### 3.1 Data sources

**Figure 1:**
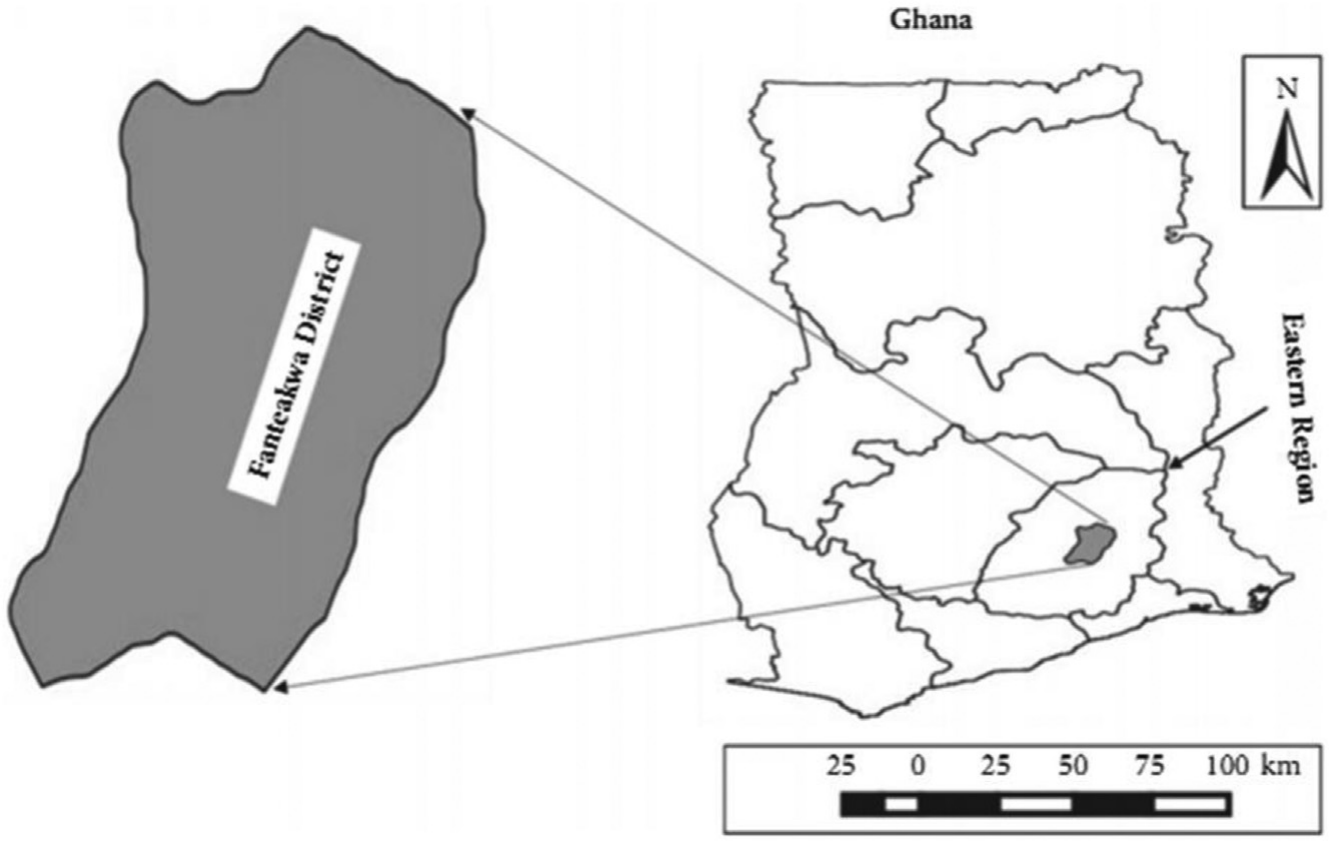
A map of Ghana showing the location of Fanteakwa District.

This study was carried out in the Fanteakwa District of the Eastern region of Ghana (see Figure 1). The district is located at longitudes 0° 10 East and latitudes 6° 15’ North. The vegetation comprises the savanna scrub and wet semi-deciduous rain forest with bimodal rainfall. The mean annual rainfall varies between 1500mm and 2000mm, while the population of the district is about 121,714 people (Ghana Statistical Service [GSS], 2016). Cassava is one of the staples in the area, with over 50% of the population engaged in cassava cultivation (GSS, 2016). A multistage sampling technique was employed in getting the sample for the study. The first stage involved the purposive selection of five cassava producing communities in Fanteakwa district, following information obtained from the district assembly. In the second stage, a list of smallholder cassava farmers was obtained from the district office for each of the five cassava-producing communities, to form the sampling frame. The final step was a simple random sampling of 60 smallholder cassava farmers from each community through the random number technique. Random numbers were assigned to each farmer on the list, and the first 60 cassava farmers were selected for the survey. This was done for each of the five communities. To cater to problems such as incompletely filled questionnaires, 5 more respondents were interviewed in each community, but the final sample size used for analysis was 300 smallholder cassava farmers.

Data was collected on farmers’ socio-economic and farm-specific characteristics such as age, gender, education, household size, farm size, output, input quantities, extension access, membership in farmer-based groups, off-farm income, and experience in cassava cultivation. The semi-structured interviews were conducted with the help of agricultural extension agents who served as enumerators. We obtained informed consent from each of the farmers before their interview.

### 3.2 Analytical framework

#### 3.2.1 The stochastic frontier model

This approach was proposed independently by Aigner et al. (1977) and Meeusen and van den Broeck (1997). The underlying assumption of this method is that firms often do not achieve their potential due to the presence of inefficiencies (Aigner et al., 1977). Given a multiple input and a single output scenario, the relationship is expressed as:

(1) $$Y_{i}=f\left(X_{i},\beta \right)+\;\varepsilon_{i}$$

where $Y_{i}$ is the scaler output of the ith firm; $X_{i}$ is a vector of inputs used by the ith firm; β is a vector of parameters to be estimated. The model assumes the error term $\varepsilon_{i}$ to be composed of two components namely; the random error component which captures factors beyond the control of the firm but affects output as well as other statistical noises; and the inefficiency component which captures those factors the firm can control. The stochastic frontier model is specified as:

(1a) $$Y_{i}=f\left(x:\beta \right).\text{exp}\left(V_{i}-U_{i}\right)$$

f(x:β) represents the production function; x represents the vector of inputs; $V_{i}$ is the random error component; while $U_{i}$ is the inefficiency component of the error term that captures the amount by which the firm is producing their optimal level or frontier. $V_{i}$ is assumed to be independently and identically distributed from $U_{i}$ (Jondrow et al., 1982). From the stochastic frontier, the technical efficiency of ith firm is given by:

(2) $$TE_{i}=\frac{Y_{i}}{Y_{i}^{*}}=\frac{f\left(X_{i};\beta \right)exp\left(Vi-U_{i}\right)}{f\left(X_{i};\beta \right)\text{exp}\left(Vi\right)}=\exp\left(-U_{i}\right)$$

Following Jondrow et al. (1982), the conditional mean of $U_{i}$ is given as:

(2a) $$E\left(U_{i}|\varepsilon_{i}\right)=\sigma_{*}^{2}\left[\frac{f^{*}\left(\varepsilon_{i}\lambda /\sigma \right)}{1-F^{*}\left(\varepsilon_{i}\lambda /\sigma \right)}-\frac{\varepsilon_{i}\lambda }{\sigma }\right]$$

where $\lambda =\sigma_{u}/\sigma_{v}$; $\sigma =\sqrt{\sigma_{u}^{2}+\sigma_{v}^{2}}$; $\sigma_{*}^{2}=\sigma_{v}^{2}\sigma_{u}^{2}/\sigma^{2}$; and $f^{*}$ represents the standard normal density function and $F^{*}$, the distribution function which is evaluated at $\left(\varepsilon_{i}\lambda /\sigma \right)$ (Jondrow et al., 1982).

The production function can follow either the Cobb-Douglas functional form or the translog functional form. The translog functional form is generally specified as (Kymn and Hisnanick 2001):

(3) $$lnY=\beta_{0}+\sum_{i=1}^{n}\beta_{i}lnX_{i}+0.5\sum_{i=1}^{n}\sum_{j=1}^{n}\beta_{ij}lnX_{i}lnX_{j}$$

where Y is the real output, $X_{i}$ is the ith factor of production and $X_{j}$ is the jth factor of production. Differentiating Eq. (5) with respect to $X_{i}$ produces the marginal product:

(3a) $$\frac{\partial Y}{\partial X_{i}}=\beta_{0}+\sum_{i=1}^{n}\beta_{ij}\cdot lnX_{i}$$

Eq. (3a) is the functional form of the Cobb-Douglas production function (Pavelescu, 2011).

##### 3.2.1.1 Empirical model specification

In this study, five factors of production were considered. Thus, the production function is empirically specified as:

(4) $$lnY=\beta_{0}+\sum_{i=1}^{5}\beta_{i}lnX_{i}+0.5\sum_{i=1}^{5}\sum_{j=1}^{5}\beta_{ij}lnX_{i}lnX_{j}+\left(V_{i}-U_{i}\right)$$

where $Y_{i}$ represents the cassava output of farmer i in kilograms; $X_{ij}$ represents five inputs (j=1,2,…,5) used by the *i*th farmer, i.e., labor, cassava stem cuttings (seed), land, herbicides and pesticides. All the quantities were standardized by dividing each quantity by its respective mean and then log-transformed.

Following Wang and Schmidt (2002), the effects of covariates on technical efficiency can be estimated in a one-step procedure. It is done by imposing the covariates in the estimation of the technology and the farmer's efficiency levels. Thus, the factors influencing the technical inefficiency is captured in the inefficiency model specified as:

(4a) $$\mu_{i}=\delta_{0}+\sum_{l=1}^{n}\delta_{l}Z_{ik}$$

where $\mu_{i}$ is the inefficiency component of the stochastic frontier and $Z_{ik}$ is the vector of the *l*th farm-level, socio-economic and institutional factor hypothesized to influence the *i*th farmer's technical inefficiency (k=1,2,…,n), while *δ* is the vector of unknown parameters to be estimated. The $Z_{ik}$ variables incorporated in the model included gender of the household head, membership in a farmer-based organization, proximity to the farmland, land ownership, extension access, education level, and experience in cassava farming. Incorporating education, extension, and experience in cassava farming was based on the findings of previous studies (Al-Hassan, 2012; Bempomaa and Acquah, 2014; Abdallah, 2016). These studies found a negative relationship between the factors and technical inefficiency. Land tenure was also incorporated in the model as one of the explanatory variables since there are different levels of land use by owners and tenants (Al-hassan, 2012). Inclusion of the location of the farm in the model was based on the findings of Kuwornu et al. (2013) that, differences in zones (locations) may lead to differences in management practices or climatic factors, thus influences the inefficiency of maize farmers in Ghana.

##### 3.2.1.2 Hypothesis tests

The following hypotheses were tested through the generalized likelihood ratio test.

1 $H_{0}:\beta_{jk}=0$; the coefficients of the interaction terms in the translog production function are zero, thus the Cobb-Douglas production function best fits the data
2 $H_{0}:\gamma =\delta_{1}=\delta_{2}\ldots =\delta_{k}=0$; Inefficiencies are absent at every level

The test statistic is calculated as:

(5) $$LR=-2\left[ln\left(\frac{H_{0}}{H_{1}}\right)\right]=-2\left[lnH_{0}-lnH_{1}\right]$$

Where $lnH_{0}$, and $lnH_{1}$, are the log-likelihood values for the null and alternative hypothesis, respectively (see Table 1).

**Table 1:** Hypotheses validation.

Null Hypothesis	Likelihood ratio	Critical value	Decision
$H_{0}:\beta_{\mathrm{jk}}=0$	98.26	28.49	Reject $H_{0}$
$H_{0}:\gamma =\delta_{1}=\delta_{2}\ldots =\delta_{k}=0$	147.39	36.94	Reject $H_{0}$

Critical values were obtained from Tabl 1 of Kodde & Palm (1986).

#### 3.2.2 The endogenous switching regression (ESR) model

A major issue with impact assessment is that of selection bias (Coulibaly et al., 2017; Bocher et al., 2017; Teklewold et al., 2013; Shiferaw et al., 2014). The ESR model uses conditional means to estimate actual and counterfactual outcomes, and also controls for both observed and unobserved heterogeneities. It is estimated in two steps. Step one involves using the probit model to estimate the factors influencing farmers’ decision to access credit from RCBs. The second step involves the specification of two linear regressions; one for accessors, and the other for non-accessors of RCB credit (Shiferaw et al., 2014; Khonje et al., 2015; Khanal et al., 2018).

In this study, access to RCB credit is assumed to be a binary choice where the farmer decides by weighing the expected benefits of accessing credit from rural banks as against not accessing. Thus, in stage 1 of the ESR model, we define a latent variable $P_{i}^{*}$, which observes the potential benefits of accessing credit from RCBs. The model is specified as:

(6) $$P_{i}^{*}=\alpha Z_{i}+\varepsilon ;\;\text{with }P_{i}=\left\{ \begin{array}{ll} 1 & if\;P_{i}^{*}>0\\ 0, & if\;otherwise \end{array}\right.$$

where $\;Z_{i}$ is an nxj matrix of household and farm-level characteristics that influences a household's decision to access credit from RCB and α is a jx1 vector of parameters to be estimated; $\varepsilon_{i}$ is a nx1 vector of normally distributed error terms.

The outcome equation is estimated separately for each regime of credit access as specified below:

(7a) $$TE_{1}=X_{1}\beta_{i}+\varepsilon_{1},\;if\;P_{i}=1$$

And

(7b) $$TE_{0}=X_{0}\beta_{0}+\varepsilon_{0},\;if\;P_{i}=0$$

$TE_{1}$ and $TE_{0}$ are the estimated technical efficiency scores for credit accessors and non-accessors, respectively; $X_{1}$ and $X_{0}$ are nxk matrices of covariates. $\beta_{i}$ and $\beta_{0}$ are parameters to be estimated, and $\varepsilon_{1}$ and $\varepsilon_{0}$ are nx1 vectors of normally distributed error terms with a zero mean and a non-zero covariance matrix:

(8) $$cov\left(\varepsilon ,\varepsilon_{1},\varepsilon_{0}\right)=\left[\begin{array}{lll} \sigma_{\varepsilon }^{2} & \sigma_{\varepsilon \varepsilon_{1}} & \sigma_{\varepsilon \varepsilon_{0}}\\ \sigma_{\varepsilon_{1}\varepsilon } & \sigma_{\varepsilon_{1}}^{2} & .\\ \sigma_{\varepsilon_{0}\varepsilon } & . & \sigma_{\varepsilon_{0}}^{2} \end{array}\right]$$

where $\sigma_{\varepsilon }^{2}=var\left(\varepsilon \right)$, $\sigma_{\varepsilon_{1}}^{2}=var\left(\varepsilon_{1}\right)$, $\sigma_{\varepsilon_{0}}^{2}=var\left(\varepsilon_{0}\right)$, $\sigma_{\varepsilon \varepsilon_{1}}=cov\left(\varepsilon ,\;\varepsilon_{1}\right)\;$, $\sigma_{\varepsilon \varepsilon_{0}}=cov\left(\varepsilon ,\;\varepsilon_{0}\right)$. Since $TE_{1}$ and $TE_{0}$ cannot be observed concurrently, the covariance between $\varepsilon_{1}$ and $\varepsilon_{0}$ is undefined (Ngoma, 2018). The non-zero covariance may result from the presence of some unobservable characteristics that influence the decision to access credit from RCBs and at the same time influence technical efficiency. The expected errors conditional on access to credit are non-zero and expressed as (Asfaw et al., 2012; Khonje et al., 2015):

(9a) $$E\left(\varepsilon_{1}|P_{i}=1)=E(\varepsilon_{1}|\varepsilon >-\alpha Z_{i}\right)=\sigma_{\varepsilon_{1}\varepsilon }\left[\frac{\theta (Z_{i}\alpha |\sigma )}{\phi (Z_{i}\alpha |\sigma )}\right]=\sigma_{\varepsilon_{1}\varepsilon }\lambda_{1}$$

(9b) $$E\left(\varepsilon_{0}|P_{i}=0)=E(\varepsilon_{0}|\varepsilon \leq -\alpha Z_{i}\right)=\sigma_{\varepsilon_{0}\varepsilon }\left[\frac{-\theta (Z_{i}\alpha |\sigma )}{1-\phi (Z_{i}\alpha |\sigma )}\right]=\sigma_{\varepsilon 0\varepsilon }\lambda_{0}$$

where θ and ϕ are the probability density function (PDF) and the cumulative density function (CDF), respectively; $\lambda_{1}$ and $\lambda_{0}$ represent the ratio of θ and ϕ evaluated at $\alpha Z_{i}$, and is referred to as the inverse mills ratio (IMR). It provides the correlation between access to credit and technical efficiency. The presence of selection bias is confirmed if the coefficients of correlation between the selection equation and the outcome equations are statistically significant. To control for selection bias, $\sigma_{\varepsilon_{1}\varepsilon }\lambda_{1}$ and $\sigma_{\varepsilon_{0}\varepsilon }\lambda_{0}$ are incorporated into the outcome Eqs. (7a) and (7b), respectively, and estimated using the full information maximum likelihood (FIML) estimator (Amanullah et al., 2019). The outcome equations are, therefore, re-specified as:

(9c) $$TE_{1}=X_{1}\beta_{1}+\sigma_{\varepsilon_{1}\varepsilon }\lambda_{1}+\mu_{1\;},\;for\;P_{i}=1$$

and

(9d) $$TE_{0}=X_{0}\beta_{0}+\sigma_{\varepsilon_{0}\varepsilon }\lambda_{0}+\mu_{0},\;for\;P_{i}=0$$

In this study, we employed the FIML approach proposed by Lokshin and Sajaia (2004). This method estimates (6) (9c) and (9d) simultaneously.

### 3.2.2.1 Specification of actual and counterfactual outcomes

Actual outcomes refer to the expected technical efficiency of farmers who accessed credit, and counterfactual outcomes explain the expected technical efficiency of farmers who accessed RCB credit had they not accessed. The conditional expectations for the different outcomes are specified below as:

(10a) $$E\left(TE_{1}|P_{i}=1\right)=X_{1}\beta_{1}+\sigma_{\varepsilon_{1}\varepsilon }\lambda_{1}$$

(10b) $$E\left(TE_{0}|P_{i}=0\right)=X_{0}\beta_{0}+\sigma_{\varepsilon_{0}\varepsilon }\lambda_{0}$$

(10c) $$E\left(TE_{0}|P_{i}=1\right)=X_{1}\beta_{0}+\sigma_{\varepsilon_{0}\varepsilon }\lambda_{1}$$

(10d) $$E\left(TE_{1}|P_{i}=0\right)=X_{0}\beta_{1}+\sigma_{\varepsilon_{1}\varepsilon }\lambda_{1}$$

Eqs. (10a) and (10b) are the expected outcomes (technical efficiencies) for accessors and non-accessors of RCB credit, respectively. Eq. (10c) is the expected technical efficiency of non-accessors, had they accessed (counterfactual for non-accessors). Eq. (10d) is the expected technical efficiency of accessors had they not accessed (counterfactual for accessors).

The average treatment effect on the treated (ATET) is the difference between the actual outcome for accessors and the counterfactual outcome for non-accessors (that is (10a) minus (10c). It captures the effect of access to RCB credit on the technical efficiency of farmers that accessed. It is expressed as:

(10e) $$ATET=E\left(TE_{1}|P_{i}=1)-\;E(TE_{0}|P_{i}=1\right)$$

The average treatment effect on the untreated (ATEU) is calculated in the same light. It is the difference between the actual expected outcomes for non-accessors and the counterfactual outcome for accessors (that is (10d) minus (10b)). That is the difference between the technical efficiency of accessors had they not accessed; and the technical efficiency of non-accessors for not accessing. It is expressed as:

(10f) $$ATEU=E\left(TE_{1}|P_{i}=0)-\;E(TE_{0}|P_{i}=0\right)$$

The next parameter to calculate is the base heterogeneity effect (BHE). It is the difference between the ATET and the ATEU (Ngoma, 2018). It is specified as:

(10g) $$BHE=ATET-ATEU=E\left(D_{1}|P_{i}=1)-\;E(D_{0}|P_{i}=1\right)-\left\{\;E\left(D_{1}|P_{i}=0)-\;E(D_{0}|P_{i}=0\right)\right\}$$

## 4 Results

### 4.1 Descriptive statistics

Table 2 presents the statistical differences in some of the socioeconomic characteristics of accessors and non-accessors of RCB credit. The table shows that credit accessors are statistically different from non-accessors in terms of age and experience. It suggests that accessors are older and more experienced in cassava cultivation than non-accessors with average ages of 44 and 43, respectively. The average number of years of experience in cassava cultivation is 12 and 9 years, respectively. There were, however, no statistically significant differences between the two groups of cassava farmers, in terms of household size, yield, income from off-farm activities, farm size, proximity to farmland, and proximity to the bank.

**Table 2:** Test of mean differences between RCB credit accessors and non-accessors.

Variable	Total	Accessors (1)	Non-Accessors (2)	Mean Diff (2)–(1)
**Demographic characteristics**				
Age	44 (0.605)	44 (0.976)	43 (0.765)	-1.580^a^∗ (1.224)
Household size	4.797 (2.230)	4.770 (0.197)	4.816 (0.170)	0.046^a^ (0.261)
Gender of Household head				
Male	217	96	121	1.631^b^
Female	83	30	53	
Experience	10.610 (7.610)	12.600 (9.240)	9.170 (5.790)	-3.420^a^∗∗∗ (0.870)
Education				
At least Primary	203	88	115	0.471^b^
No Formal Educ	97	38	59	
Off-farm income	595.240 (629.280)	594.390 (555.740)	595.850 (679.160)	1.450^a^ (73.740)
**Farm-level characteristics**				
Yield	5045.040 (7364.210)	4281.330 (9120.150)	5598.070 (3686.600)	1316.740^a^ (859.510)
Farm size	3.638 (2.810)	3.505 (2.973)	3.735 (2.689)	0.231^a^ (0.329)
Variable	Pooled	Accessors	Non-Accessors	Mean diff
**Institutional characteristics**				
Proximity to Bank	4.138 (7.712)	3.831 (7.079)	4.360 (8.153)	0.528^a^ (0.903)
Proximity to farmland	2.754 (1.813)	2.738 (1.738)	2.765 (1.871)	.027^a^ (0.212)
FBO Membership				
Yes	95	51	44	7.791^b^∗∗∗
No	205	75	130	
Extension access				
Yes	149	69	80	2.256^b^
No	151	57	94	
Land tenure				
Owned	81	44	37	6.854^b^∗∗∗
Not Owned	219	82	137	
Savings				
Yes	151	72	79	4.040^b^∗∗
No	149	54	95	

Note: ∗p < 0.1 ∗∗p < 0.05 ∗∗∗p < 0.01 ^a^one-tail t-test ^b^two-tail t-test ^c^chi2 statistic; standard errors in parentheses.

Table 2 further shows that there is a statistically significant difference between accessors and non-accessors, in terms of membership in farmer-based organizations. There is also a significant difference in terms of land tenure as well as ownership of savings account. However, there was no statistical evidence of any differences between accessors and non-accessors in terms of the gender of the household head, access to extension services, and educational level of the household head.

The above statistics, however, do not depict the exact impact of access to credit on the technical efficiency of cassava farmers in the Fanteakwa District. Conclusions on the impact of access to RCB credit on technical efficiency based on the above differences will be biased. Policy recommendations should, therefore, not be based on them.

### 4.2 Technical efficiency of cassava farmers

#### 4.2.1 Estimates of the translog production function

The results of the maximum likelihood estimation of the translog production function (equation 6) are presented in Table 3. The main objective of this study is to measure the impact of access to credit from RCBs on technical efficiency, therefore, discussions on the frontier estimates will be brief.

**Table 3:** Maximum likelihood estimation of the translog production function.

Inputs	Coefficient	Robust Std. Err.	t-value
lnPesticides	0.152	0.114	1.330
lnHerbicides	0.314	0.174	1.810∗
lnLabor	0.290	0.125	2.320∗∗
lnFarm	0.513	0.103	5.000∗∗∗
lnSeed	0.242	0.092	2.640∗∗∗
lnLabor2	0.957	0.391	2.450∗∗∗
lnSeed2	0.080	0.166	0.480
lnFarm2	-0.019	0.187	-0.100
lnPest2	-0.085	0.122	-0.700
lnHerb2	1.123	0.382	2.940∗∗∗
SeedxLabor	-0.510	0.336	-1.520
FarmxLabor	-0.107	0.475	-0.230
FarmxSeed	0.282	0.162	1.740∗
SeedxPesticides	0.585	0.194	3.020∗∗∗
PestxLabor	1.131	0.501	2.260∗∗
PestxFarm	0.025	0.383	0.060
HerbsxLabor	-0.560	0.572	-0.980
HerbxFarm	0.040	0.327	0.120
HerbxSeed	-0.521	0.335	-1.550
HerbxPest	-0.290	0.345	-0.840
Constant	1.054	0.181	5.830∗∗∗

Note: ∗p < 0.1 ∗∗p < 0.05 ∗∗∗p < 0.01.

The model produced a final log-likelihood value of -192.501 with a likelihood ratio chi-square of 303.50 (*df = 20)*, significant at the 1 percent. This suggests that the model wholly fits the data better than a model with no predictors. The coefficients of the first-order derivatives of the translog production function are interpreted as the partial output elasticities. The estimated first-order coefficients are positive and imply that the monotonicity condition of a rational production function is satisfied (Otieno et al., 2011). The first-order coefficients for farmland (*lnFarm)* and cassava stem cuttings *(lnSeed)* are all positive and significant at 1 percent. The coefficient for labor (*lnlabor)* is significant at 5 percent and that of herbicides (*lnHerbicides)* at 10 percent. The first order coefficient of pesticides (*lnPesticides)* is statistically insignificant (Table 3). The coefficients of *lnHerbicides, lnLabor, lnFarm* and *lnSeed* are 0.314, 0.290, 0.513 and 0.242, respectively (Table 3). These imply that a 1 percent increase in the input quantities would lead to approximately 0.314, 0.290, 0.513, and 0.242 percent increases in output, respectively, holding the levels of the other factors of production constant.

The coefficients of the squared terms of the inputs represent the second-order derivatives. A positive coefficient of the squared term suggests that the marginal physical product (MPP) would increase with additional units of the variable input and vice versa, *ceteris paribus* (Bai et al., 2019). Thus, the coefficients of the squared of farm labor (*lnLabor2* = 0.957) suggest that the current level of labor is not optimal and that the MPP of labor continues to rise with every increase in the quantities of labor. Similarly, the estimated coefficient of the squared of seed (*lnSeed2 =* 0.080) and the squared of herbicides (*lnHerb2 =* 1.123) suggests that increases in the current quantity of stem cuttings, and herbicides will lead to increases in their respective MPP, holding the quantities of the other factors unchanged. The negative coefficients point to the fact that the current level of the variable factor is above the maximum level. Therefore, any further increase in the levels of the inputs, holding other things constant, will cause the MPP to fall, thereby leading to detrimental effects on yields.

The coefficient of the cross terms (the interaction terms) suggests whether the interacted inputs are complements or substitutes to each other (Bai et al., 2019; Abdulai et al., 2018). In this study, the interaction between seed and pesticides produced a positive coefficient (0.585) statistically significant at 1 percent. It suggests that seeds and pesticides are complements. It also indicates that a simultaneous increase in the quantities of stem cuttings (seed) and pesticides will increases yields, *ceteris paribus*. The interaction between pesticides and labor also produced a positive coefficient, which implies that labor and pesticides are complements. The interaction between farmland and seed also generated a positive coefficient suggesting that farmland and cassava stem cuttings are complements. Seed and labor, however, had a negative coefficient. It implies that any simultaneous increases in the quantities of stem cuttings and labor will have reducing effects on yields, given that the levels of the other inputs remain constant.

#### 4.2.2 Distribution of technical efficiency score among cassava farmers

**Figure 2:**
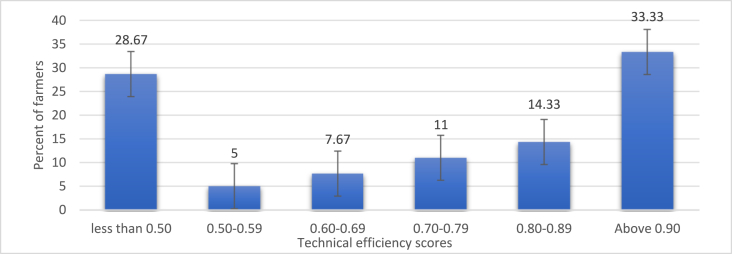
Distribution of technical efficiency scores of cassava farmers in Fanteakwa district. Source: Field survey (2019).

The minimum and maximum technical efficiencies (TE) recorded were 19.1 percent and 99.4 percent, respectively, with the average TE score being 70.5 percent. About 33 percent of the farmers recorded technical efficiencies in the range of 90–99 percent. The TE score range of 50–59 percent recorded the least number of farmers (5 percent of the farmers). Figure 2 presents the distribution of estimated TE scores among cassava farmers in the district.

#### 4.2.3 Determinants of technical inefficiency

Table 4 presents the maximum likelihood estimation results of the technical inefficiency model (equation 4a). A negative (positive) coefficient suggests that the variable has a positive (negative) effect on technical efficiency. Among the farmer characteristics included in the model, the educational status of the farmer (the household head) is statistically significant with a negative coefficient. Also, the coefficient of proximity to farmland is negative and significant at 10 percent. Similarly, the results show that access to extension services negatively influences technical inefficiency (positively influences TE).

**Table 4:** Determinants of technical inefficiency amongst Cassava farmers in Fanteakwa district.

Factors	Coefficient	Robust Std Err	t-value
Constant	1.780	0.557	3.200∗∗∗
**Farmer characteristics**			
Gender (1 = Male)	-0.258	0.173	-1.490
Education	-0.264	0.150	-1.760∗
Experience	-0.008	0.011	-0.710
Income from off-farm activity	-0.100	0.097	-1.030
Proximity to farmland	-0.227	0.133	-1.710∗
**Farm-level factors**			
Ahomahomasu	0.797	0.350	2.280∗∗
Begoro	0.859	0.487	1.770∗
Feyiase	-6.928	3.936	-1.760∗
Akoradarko	-0.181	0.384	-0.470
**Institutional factors**			
FBO membership(1 = Yes)	-0.246	0.193	-1.270
Extension Access (1 = Yes)	-0.239	0.117	-2.050∗∗
Land tenure (1 = Owned)	0.006	0.110	0.050

Note: ∗p < 0.1 ∗∗p < 0.05 ∗∗∗p < 0.01.

The coefficients for Ahomahomasu and Begoro were both positive and significant at 5 percent and 10 percent, respectively, using Obuoho as the reference community. The results imply that cassava farmers in Ahomahomasu and Begoro are less technically efficient than those in Obuoho. The coefficient for Feyiase produced a negative coefficient estimate (at *p < 0.1*), suggesting that cassava farmers in the Feyiase community are more technically efficient. These differences could be ascribed to differences in farm management practices as well as climatic and edaphic factors. The social capital variable (*FBO membership*) met the *a priori* expectation of a negative effect on technical inefficiency, although it is statistically insignificant. The negative coefficient means membership in farmer-based groups helps to improve the TE of the farmers.

### 4.3 RCB credit access and technical efficiency of smallholder cassava farmers

#### 4.3.1 Maximum likelihood estimation of the ESR model

Table 5 present the results of stage 1 of the FIML estimation of the ESR model. The parameter, rho_1, is negative and statistically significantly different from zero. It confirms that self-selection occurred in RCB credit access. The likelihood ratio test for independence produced a chi-square value of 2.730 statistically significant at 10%. The implication is that the three Eqs. (8, 9c, and 9d) are not mutually exclusive, thus should not be estimated separately, thereby justifying the use of the ESR model. The results from the estimation of Eqs. 9c and 9d (the OLS estimation of the determinants of TE for each of the two groups) have been omitted due to space limitations. It is, however, worthy to note that the discussion of those results is similar to the discussions in section 4.2.3.

**Table 5:** Determinants of farmer's decision to access RCB credit.

Factors	Coef.	Std. Err	t-value
Constant	2.873	1.187	2.420∗∗
**Demographic Factors**			
Gender (1 = Male)	-0.031	0.295	-0.100
Education (1 = Formal Education)	0.096	0.120	0.800
Age	0.023	0.015	1.500
Household size	-0.028	0.060	-0.480
Off-farm Income	-0.073	0.140	-0.520
Savings	0.666	0.325	2.050∗∗
**Farm-level characteristics**			
Farm size	-0.079	0.045	-1.740∗
Farm Location (1 = Begoro)	-0.086	0.327	-0.260
**Institutional Factors**			
FBO membership (1 = Yes)	1.088	0.342	3.180∗∗∗
Extension access (1 = Yes)	0.700	0.290	2.420∗∗∗
Land tenure (1 = owned)	0.223	0.300	0.740
Proximity to Bank	-1.369	0.156	-8.770∗∗∗
Lns_0	-1.627	0.302	-5.390∗∗∗
Lns_1	-1.513	0.214	-7.080∗∗∗
Rho_0	0.507	0.339	-1.500∗∗∗
Rho_1	-0.212	0.270	-0.780∗∗∗
LR test chi^2^ (1)			2.730∗

Note: ∗p < 0.1 ∗∗p < 0.05 ∗∗∗p < 0.01.

The result from the first stage of the ESR shows that among the demographic and socioeconomic factors that were included in the model, having a savings account with the bank (*savings*) met the *a priori* expectation of a positive effect on farmers’ decision to access credit from the RCBs and is statistically significant (Table 5). Gender of the household head (*Gender)* and educational status of the household head (*Education)* met their respective *a priori* expectations but are statistically insignificant. However, contrary to our *a priori* expectation, the coefficient of farm size is negative and significant at 10 percent. It means that the larger the farm, the lesser likelihood that a smallholder cassava farmer will access credit from RCBs.

Among the institutional factors that were hypothesized to influence farmers' decision to access credit from RCBs, access to agricultural extension services met the *a priori* expectation of a positive effect on farmers' access to credit from RCBs (*p < 0.01*). The implication is that farmers who access agricultural extension agents are more likely to access credit from RCBs in the District. Proximity to the bank also met the *a priori* expectation of a negative effect on farmers' access to credit from RCBs. It suggests that farmers who stay farther away from the RCB are less likely to access it. In other words, living closer to the bank increases farmers’ likelihood of going for credit from RCB. Membership in farmer organizations also met the *a priori* expectation of a positive effect on RCB credit access. The positive coefficient means that a smallholder cassava farmer will be more inclined to access credit from RCBs if the farmer joins a farmer group.

#### 4.3.2 Impact of RCB credit access on technical efficiency

Table 6 presents the estimates of the expected TE scores given the actual and counterfactual scenarios. Both the accessors and non-accessors of RCB credit face two possible decisions; to access or not to access. The diagonal cells (a and d) present the expected technical efficiencies under the actual scenarios. The off-diagonal cells (b and c) show the expected technical efficiencies under the counterfactual scenarios.

**Table 6:** Impact of access to RCB credit on the technical efficiency of cassava farmers.

Sub-Sample	N	Decision		Treatment effects	
		To access	Not to access		
Accessors	126	(a) 0.718 (0.116)	(b) 1.132 (0.089)	ATET	0.483∗∗∗ (0.009)
Non-accessors	174	(c) 0.235 (0.153)	(d) 0.707 (0.160)	ATEU	0.425∗∗∗ (0.008)
				BHE	0.058∗∗∗ (0.01)

Note: ∗∗∗p < 0.01; ATET (a-c), ATEU (b-d) and BHE (ATET-ATEU) are average treatment effects on the treated, average treatment effects on the untreated, and base heterogeneity effect, respectively.

The expected average TE of smallholder cassava farmers who accessed RCB credit (a), is 0.718 and that of non-accessors (d) is 0.707 (Table 6). Cells (a) and (d) represent the expected average TE scores of cassava farmers in the actual scenario. In the counterfactual scenario, the expected average TE score of RCB credit accessors, assuming they had not accessed (b), is 1.132, and that of non-accessors assuming they accessed (c) is 0.235. From Table 6, the average treatment effect on the treated (ATET), which is the difference between cells (a) and (c), is 0.483. The treatment effect on the untreated (ATEU), obtained by the difference between cells (b) and (d), is 0.425. The difference between the ATET and the ATEU is the base heterogeneity effect (BHE), which is 0.058. All the treatment effects are statistically significant at 1 percent.

## 5 Discussions

### 5.1 Technical efficiency of cassava farmers

The average TE score of 70.5 percent suggests that cassava farmers in Fanteakwa districts produce below their potential output, therefore, are technically inefficient. The implication is that farmers can increase yield by 29.5 percent without increasing the current level of inputs they use. Furthermore, the positive contributions of farmland, cassava stem cuttings, and labor to cassava yield, signal the importance of such factors. Therefore, the need to consider both material and human factors in cassava development programs. Agricultural development programs aimed at providing farmers with planting materials and fertilizer, such as the Planting for Food and Jobs (PFJ) program and the Fertilizer Subsidy Program (GFSP), can be restructured to include cassava as one of the priority crops. Sustainable investments made on agricultural technologies will lead to improvements in the TE of cassava farmers. This result is corroborated by Afrin et al. (2017) who analyzed the impact of financial inclusion on the technical efficiency of paddy farmers in Bangladesh and reported that land, labor, and seeds positives contributed to output. It is also consistent with Abdul-Kareem and Isgin (2016) who estimated the technical efficiency of cassava producers in Northern Ghana.

The negative coefficient of formal education in the inefficiency model suggests that formal education reduces farmers’ technical inefficiency (that is, it improves TE). The implication is that farmers who have attained at least primary formal education tend to be relatively more efficient in their resource use. This result may be attributed to the fact that improvement in human capital is an enabling factor for efficient resource utilization among rural households. This finding is in line with Zakaria et al. (2019) who examined the impact of financial development on agricultural development in South Asia and found that increase in human capital leads to improvements in agricultural productivity. It is also consistent with Wongnaa and Awunyo-Vitor (2018) who investigated the factors that affect the profit efficiency of smallholder maize farmers in Ghana and found formal education to improve the profit efficiency of the farmers. This result suggests that educational programs targeted at rural non-formally educated farmers will contribute immensely to the human capital development of such farmers, consequently leading to improvements in their TE.

The negative coefficient of proximity to farmland implies that farmers who stay farther from their farmlands are less technically inefficient (that is, they are more technically efficient) vis-a-vis farmers who live closer to their farmlands. This finding is surprising as the *a priori* expectation was that farmers who stay closer to their farmlands would have easy and timely access to the farm. Staying closer to the farmland makes it easier for the farmer to carry out the best farm management practices since the farmer incurs little or no cost when accessing the farm. This result contradicts Zeng et al. (2018) who found an inverse relationship between the distance from the farmland to town and the TE of grain farmers in China. It also contradicts the findings of Danquah et al. (2019) who reported that in Ghana, the farther away maize farmers are from their farmlands, the more technically inefficient they are.

Agricultural extension agents are a vital source of agricultural information for farmers in the rural parts of Ghana. The negative coefficient of extension access in this study implies that smallholder cassava farmers with access to agricultural extension services are less technically inefficient collated with farmers without access to extension services. This finding is in line with that of Ali and Awade (2019), who found that access to extension services significantly improves the productivity of credit-constrained soybean farmers in Togo. Enhancements in the scope and delivery of agricultural extension services would translate into significant improvements in the TE of farmers in the district. Setting up more programs like the flagship Ghana Extension Systems Strengthening Project (GESSiP) will be a step in the right direction to improving extension service delivery in the country.

Although the social capital variable, FBO membership, was statistically insignificant for farmers in the Fanteakwa district, the negative coefficient supports the assertion that farmers who belong to farmer groups get the chance to learn from their fellow farmers. They learn about the latest output-enhancing technology and the best farm management practices, among other things, thereby making them less technically inefficient compared to farmers who are not members. Therefore, visible support from the government to farmer organizations will encourage more farmers to join such groups. The long-run effect of this is improvements in farmers’ resource use efficiency. This finding is supported by Anang et al. (2017), who also found a negative relationship between FBO membership and TE of rice farmers in Northern Ghana.

### 5.2 RCB credit access and technical efficiency of smallholder cassava farmers

The statistically significant rho from the ESR model in Table 5 signifies the presence of self-selection in accessing RCB credit. On average, farmers who accessed credit from RCBs are about 48 percent more technically efficient as can be seen from the value of ATET in Table 6. Furthermore, the value of ATEU suggests that on average, farmers who accessed RCB credit would have been about 43 percent less technically efficient had they not accessed credit from RCBs. The treatment effects mentioned so far help to account for the selection bias arising from the symmetrical differences between accessors and non-accessors (Khanal et al., 2018). The positive contribution of access to RCBs to the TE of the farmers is reinforced by the base heterogeneity effect (BHE). The positive and statistically significant BHE suggests that accessors and non-accessors are significantly different. It also confirms the presence of some unobserved heterogeneities that make accessors more technically efficient than non-accessors. The BHE also suggests that the effect of access to credit from RCBs is significantly larger for farmers who accessed than farmers who did not access. These results are similar to that of Khanal et al. (2018) who examined the impact of farmers' climate change adaptation strategies on yields and found that adaptation strategies employed increased yields significantly. It follows those improvements in the TE of smallholder cassava farmers can be achieved with increased engagement between the farmers and RCBs. The engagement is in terms of the provision of credit facilities on the part of RCBs. The improvements in TE owing to the access to credit from RCBs, observed in this study is also in line with Martey et al. (2019), who examined the impact of credit on smallholder maize farmers’ TE in Ghana and observed that the TE is positively impacted by credit.

From the results, farmers' likelihood of accessing credit from RCBs is significantly high if they own savings accounts with the bank. This finding can be attributed to the fact that most banks in Ghana run the savings-before-credit policy, which requires borrowers to have active savings account with the bank before they can be deemed eligible for credit. This result is consistent with (Twumasi et al., 2019), who also reported that savings mobilization positively impacts the credit access and the amount of credit smallholder farmers in Ghana can borrow. A sensitization program on the benefits of saving with the bank could go a long way to having more farmers save with RCBs. Extension agents provide information and training not only on the latest agricultural technology but also in farm resource management. It is, therefore, not surprising that access to extension services also improves the likelihood of farmers accessing credit from RCBs. Farmers who come into contact with extension agents learn of sources of credit for farm investments. The result observed in this study is similar to that of Djoumessi et al. (2018), who studied the determinants of smallholder vegetable farmers’ access and demand to credit in Cameroun and found that access to agricultural extension services positively influences both access to and demand for credit.

Studies have found that transaction cost influences the adoption of agricultural technology (Ugochukwu and Phillips 2018). The negative coefficient observed for *proximity to the bank* may be explained by the fact that the banks are situated in the district capital. Moreover, most of the farmers are living in communities outside the district capital. Therefore, farmers who stay farther away from the district capital are less likely to hear about the credit programs and access. Farmers who hear about the credit programs may be less motivated to access them due to the high transaction costs. The relationship observed in this study between credit access and proximity to credit source is consistent with Amanullah et al. (2019), who examined the impacts of credit constraints on rice farmers’ investment and income in Pakistan and found that a longer distance to credit source negatively influences access. Farmers who belong to farmer groups have a higher chance of hearing about RCB credit programs and have a higher probability of accessing such credits. It is because banks are more inclined to lend to groups than to individual borrowers. This result is supported by Assogba et al. (2017), who analyzed the factors influencing credit access by farmers in Benin and found that membership in farmer groups is a significant factor.

## 6 Conclusions

This paper assessed the impact of access to RCB credit on TE using farm level and household level cross-sectional data for the 2017/2018 cassava production season in the Fanteakwa district in Ghana. The stochastic frontier model was used to estimate the TE score of the cassava farmers in the District. The ESR model was used to control for self-selection into RCB credit access and generate consistent actual and counterfactual outcomes.

The results suggest that cassava farmers in the Fanteakwa district are 70.5 percent technically efficient. It implies that cassava output could be increased further by 29.5 percent by improving farmers’ efficiency. Through the Planting for Food and Jobs (PFJ) program, the government of Ghana can make available to the farmers improved cassava seeds. Also, the results indicate that the TE of farmers is knowledge-based, given the effects of extension access and membership in farmer-based groups on their resource use. It calls for policies that strengthen the farmer-based organizations and enhances the mode and scope of extension service delivery, particularly in the rural parts of the country. The gender of the household head, extension access, land ownership, and income from off-farm economic activities are the major factors that positively influence farmers to access credit from RCBs. On average, farmers who accessed credit from RCBs have higher technical efficiencies than farmers who did not. It points to the need for a more holistic information dissemination approach to increase awareness and adoption of RCB credit programs among rural-based smallholder farmers.

This study assessed the impact of RCBs on TE only at the district level. Future studies may conduct a country-level analysis to assess the overall impact of RCBs in the crop production sub-sector of Ghana. Future studies may also investigate the rates of adoption of RCB credit programs among smallholder farmers. Also, a country-level comparison of the technical efficiencies of cassava producers in SSA will shed more light on the technological differences in cassava production across the sub-region.

## Declarations

### Author contribution statement

Arnold Missiame, Rose A. Nyikal, Patrick Irungu: Conceived and designed the experiments; performed the experiments; analyzed and interpreted the data; contributed reagents, materials, analysis tools or data; wrote the paper.

### Funding statement

This work was supported by 10.13039/501100016991African Economic Research Consortium (AERC).

### Data availability statement

Data will be made available on request.

### Declaration of interests statement

The authors declare no conflict of interest.

### Additional information

No additional information is available for this paper.

## Appendix A Supplementary data

Household_Survey_Questionnaire Household_Survey_Questionnaire
