# Supplementary material for: What is the impact of rural bank credit access on the technical efficiency of smallholder cassava farmers in Ghana? An endogenous switching regression analysis
Source: Heliyon. 2021 May 21;7(5):e07102. doi: 10.1016/j.heliyon.2021.e07102 (PMC8165398; doi:10.1016/j.heliyon.2021.e07102)
Supplement: Household_Survey_Questionnaire [file mmc1.docx]

**Household Survey Questionnaire**

UNIVERSITY OF NAIROBI

WHAT IS THE IMPACT OF RURAL AND COMMUNITY BANK CREDIT ACCESS ON THE TECHNICAL EFFICIENCY OF SMALLHOLDER CASSAVA FARMERS IN GHANA? AN ENDOGENOUS SWITCHING REGRESSION ANALYSIS

QUESTIONNAIRE

| ENUMERATOR’S NAME: | DATE: |
| --- | --- |
| LOCATION: | COMMUNITY: |

**SEEKING CONSENT**

Good day Sir/Madam,

My name is _________ and I am working with the Department of Agricultural Economics of the University of Nairobi, Kenya. We are carrying out research on the impact of rural and community bank (RCB) credit access on technical efficiency of smallholder cassava farmers in Fanteakwa District of Ghana. The purpose of this study is to get views, experiences and suggestions of farmers on rural bank credit access, challenges involved, and its effect on the production of cassava. Respondents of this survey should be cassava farmers who must be at least 18 years old. You have been randomly selected and your participation in this survey is voluntary. The findings of this study will be mainly used to inform policy on improving the technical efficiency of cassava farmers. The interview will require about 45 minutes to complete. Can I proceed with the interview?

*If* ***NO,*** *enter* ***00*** *here* [ | ] *and end the interview.* ***Find a replacement household.***

*If* ***YES****, enter* ***01*** *here* [ | ] *to acknowledge that consent has been granted by the respondent.*

**SECTION A: Socio-Economic Characteristics of Cassava Farmer**

A1. Are you the head of this household? 1=Yes [ ] 0=No [ ]

A2. If NO, who is the head of the household?

*[****ENUMERATOR:*** *If the Respondent is NOT the household head, ask him/her the following questions about the household head]*

A3. What is the gender of the household head? 1 = Male [ ] 0 = Female [ ]

A4. What is the age of the household head? (*in years)*: ………………………………………

A5. What is the marital status of the household head? 0 = Single [ ] 1 = Married [ ] 2 = Separated [ ] 3 = Divorced [ ]

A6. What is the religion of the household head? 1 = Christian [ ] 2 = Muslim [ ] 3 = Traditionalist [ ] 4 = Others (specify) …………………………

A7. What is the highest education level attained by the household head? 1=Primary [ ] 2= [ ] 3=SHS/Technical [ ] 4=Tertiary [ ] 5=None [ ]

A8. What is the household size of the household head? ………………………………person(s)

| Number of adults >18yrs | | Number of adults<18yrs | | Number of Children <5yrs | |
| --- | --- | --- | --- | --- | --- |
| a. Males | b. Females | a. Males | b. Females | a. Males | b. Females |
|  |  |  |  |  |  |

**SECTION B: LIVELIHOOD SOURCES**

***i. Livelihood Sources***

B8. What are your sources of livelihood? 1=Crop farming [ ] 2=Fishing [ ] 3=Livestock keeping [ ] 4=Business [ ] 5=Petty trade [ ] 6=Wage employment [ ] 7=Salaried employment [ ] 8=Other (Specify) [ ]

B9. Please give the approximate income obtained from each source over the last ONE year.

| **Income source** | **Approximate Amount over last ONE year (In GHC)** |
| --- | --- |
| Sale of crop produce |  |
| Fish sales |  |
| Livestock sales |  |
| Sale of livestock products (e.g., milk, ghee, hides & skins, etc) |  |
| Sale of crop produce |  |
| Salary from employment ***[Multiply monthly salary by 12 to get annual amount]*** |  |
| Wage employment ***[Determine annual amount]*** |  |
| Pension/retirement benefit ***[Multiply monthly benefit by 12 to get annual amount]*** |  |
| Remittances ***[Determine annual amount]*** |  |
| Business |  |
| Other income source (Specify)……………… |  |

B10. How frequently do you receive remittances? 1=Daily [ ] 2= Weekly [ ] 3= Monthly [ ] 4=Bi-monthly [ ] 5= Yearly [ ]

B11. From whom do you usually get remittances?........................................................

***ii. Livestock Ownership***

B12. How many of the following livestock type do you have now?

| **Species** | **Total** | **Number owned** | **Number not owned** |
| --- | --- | --- | --- |
| Cattle |  |  |  |
| Sheep |  |  |  |
| Goats |  |  |  |
| Chickens/Fowls |  |  |  |

***iii. Ownership of other Assets***

B13. Indicate the number of assets owned by the household.

| **Housing type** | **Number** | **Farm transport** | **Number** | **Water storage** | **Number** |
| --- | --- | --- | --- | --- | --- |
| Grass roof/stick wall |  | Donkey cart |  | Plastic tank |  |
| Grass roof/mud wall |  | Human-drawn cart [Truck/Wheel Barrel] |  | Iron tank |  |
| Iron sheet roof/wooden wall |  | Bicycle |  | Stone tank |  |
| Iron sheet roof/mud wall |  | Motorcycle [*Okada*] |  | Other (Specify) _ |  |
| Iron sheet roof/iron sheet wall |  | Car |  | **Other Assets** |  |
| Iron sheet roof/concrete wall |  | Pickup |  | Working mobile phones |  |
|  |  | Lorry |  | Working radio |  |
|  |  | Tractor |  | Working TV |  |

**SECTION C: Crop Production**

C14. Are you involved in cassava farming? 1=Yes [ ] 2=No [ ]

C15. If YES, what is the total size of your land? ___________________________ Acres

C16. Aside cassava, which other crops did you grow last season?

| Crop | Approximate acreage with the crop last season (Acres) | Approximate quantity produced (Kgs) | Approximate quantity consumed in the household (Kgs) | Approximate quantity sold (Kgs) | Which market? |
| --- | --- | --- | --- | --- | --- |
|  |  |  |  |  |  |

C17. Do you use irrigation? 1=Yes [ ] 2=No [ ]

C18. *[IF YES]*, What area did you irrigate **last season**? ________________ acres.

C19. Where do you usually get your irrigation water from? ________________

**SECTION D: Basic information on cassava farming**

C12. How many years has the household been involved in cassava production? ………… years

B13. Please indicate the seasons in which you usually cultivate your cassava. 1= Only in the main season [ ] 2= Only in the minor season [ ] 3= In both seasons [ ]

B22. Please indicate the kind of implements employed on your farm? 1= Only simple farm tools [ ] 2= Plough [ ] 3=Ridge [ ] 4= Only 1 and 2 [ ] 5= Only 1 and 3[ ] 6= All [ ]

B23. Did you use the services of the agricultural mechanization centre for 2017/2018 farming season? 1= Yes [ ] 2= No [ ]

C28. How much does an acre of land cost if it was rented? GHS (per acre) ……………………

**SECTION F: Institutional factors**

D21. Do you belong to any farm-based group/association? 1= Yes [ ] 0= No [ ]

D22. Please indicate the most important contribution received from the group since you joined.

1= Book-keeping Training [ ] 2= Agronomic Practices [ ] 3= Credit Management [ ] 4= Others (specify)…………..….

D23. What is the type of tenure of your land?

1=Individual 2=Leasehold 3=Communal 4=Other (Specify)_____

**SECTION G: Input Use**

**Please indicate the quantity of inputs used on cassava during the MAIN season of 2017/2018 production:**

| **Input** | **Unit** | **No. of units** | **Quantity** | **Unit price** | **Source/market** |
| --- | --- | --- | --- | --- | --- |
| *Land* | Acres |  |  |  |  |
| Owned |  |  |  |  |  |
| Given |  |  |  |  |  |
| Rented |  |  |  |  |  |
| *Labor* | Manhours |  |  |  |  |
| Bush clearing |  |  |  |  |  |
| Ploughing |  |  |  |  |  |
| Harrowing |  |  |  |  |  |
| Sowing |  |  |  |  |  |
| Fertilizing |  |  |  |  |  |
| Manuring |  |  |  |  |  |
| Weeding |  |  |  |  |  |
| Irrigation |  |  |  |  |  |
| Herbicide application |  |  |  |  |  |
| Pesticide application |  |  |  |  |  |
| Harvesting |  |  |  |  |  |
| Packaging |  |  |  |  |  |
| Marketing |  |  |  |  |  |
| *Pesticides* | Litres |  |  |  |  |
| *Herbicides* | Litres |  |  |  |  |
| *Fertilizer* | Kgs |  |  |  |  |
| DAP |  |  |  |  |  |
| Etc |  |  |  |  |  |
| Sacks |  |  |  |  |  |
| *Irrigation water* | Litres? |  |  |  |  |
| *Machinery* | Machinehours |  |  |  |  |
| *Type of Cassava Stem* | Number of stems |  |  |  |  |
| Improved Stems |  |  |  |  |  |
| Locally developed-improved |  |  |  |  |  |
| Recycled locally developed-improved |  |  |  |  |  |
| Other (specify)…………. |  |  |  |  |  |
| *Cassava Variety* |  |  |  |  |  |
| AGRA Bankye |  |  |  |  |  |
| Dudzi |  |  |  |  |  |
| Abrabopa |  |  |  |  |  |
| Lamesese |  |  |  |  |  |
| Duade Kpakpa |  |  |  |  |  |
| Amansan |  |  |  |  |  |

C37. Did you apply inorganic fertilizer during the 2017/2018 farming season?

1= Yes [ ] 0= No [ ]

C38. If Yes, please indicate the type, the quantity and the cost of the inorganic fertilizer used during the 2017/2018 farming season

| Inorganic Fertilizer | Unit | Qty Used | | Price per unit (GHS) | | Total Cost (GHS) | |
| --- | --- | --- | --- | --- | --- | --- | --- |
|  |  | Major Season | Minor Season | Major Season | Minor Season | Major Season | Minor Season |
| NPK | 50kg |  |  |  |  |  |  |
| Winner | 50kg |  |  |  |  |  |  |
| Urea | 50kg |  |  |  |  |  |  |
| Sulphate of Ammonia | 50kg |  |  |  |  |  |  |
| Others | 50kg |  |  |  |  |  |  |

C39. Did you use any agro-chemical during the 2017/2018 farming season? 1= Yes [ ] 2= No [ ]

C340. If yes, please indicate the types, quantity and the total cost involved

| Inorganic Fertilizer | Unit | Qty Used | | Price per unit (GHS) | | Total Cost (GHS) | |
| --- | --- | --- | --- | --- | --- | --- | --- |
|  |  | Major Season | Minor Season | Major Season | Minor Season | Major Season | Minor Season |
| Field Fungicides | 1000ml |  |  |  |  |  |  |
| Nematicides | 1000ml |  |  |  |  |  |  |
| Weedicides | 1000ml |  |  |  |  |  |  |
| Others | 1000ml |  |  |  |  |  |  |

**SECTION H: Cassava Output**

E46. Please indicate how many kilograms of cassava you harvested during the MAIN & MINOR seasons of 2017/2018 farming season.

| Season | Approximate quantity produced (Kgs) | Approximate quantity consumed in the household (Kgs) | Approximate quantity sold (Kgs) | Unit price (GHS) | Which market? |
| --- | --- | --- | --- | --- | --- |
| Main |  |  |  |  |  |
| Minor |  |  |  |  |  |

**SECTION I: Access to Services**

E47. What is the distance from your farm to the nearest cassava buying centre? (*in kilometres)* ……….

What is the distance from your farm to the nearest market? (*in kilometres)*

E48. What is the distance from your farm to your house? (*in kilometres*) ……………

5.9C. How far is the nearest all weather road from your home? _________km

5.6C. How far is the nearest primary school from your home? _________km

5.7C. How far is the nearest hospital/health centre from your home? _________km

5.8C. How far is the nearest extension service provider from your home? _________km

5.10C. How far is the nearest water source from your home? _________km

5.8C. How far is the nearest cassava inputs provider from your home? _________km

Did you ever have contact with any extension agent in the 2017/2018 production year?

1 =Yes [ ] 0= No [ ]

If yes, how many times in: a. Major season ……………. b. Minor season………………….

What was the primary information rendered by extension agents in the 2017/2018 production year? 1= Production Information [ ] 2= Marketing Information [ ] 3= Handling and Storage Information [ ] 4= Others (specify) ……………………………….

B17. Did you receive any formal training on cassava cultivation during the 2017/2018 production year? 1= Yes [ ] 2= No [ ]

B18. If YES, where was the training conducted? 1= On the farm [ ] 2= Off farm [ ] 3= Others (Specify)…………………

B20. What was the training on? 1= Postharvest technology [ ] 2= Agronomic practices [ ] 3= Credit application processes

[ ] 4= Others (specify)………………..

**SECTION J: Awareness of RCB credit programme**

F49. Are you aware of any rural bank credit programme in your community?

1=Yes [ ] 0=No [ ]

F50. If yes, how did you become aware of the programme?

1=FBO member [ ] 2=Staff of RCB [ ] 3=Extension agent [ ] 4=Advertisement [ ] 5=Other, Please Specify………..

**SECTION K: Access to RCB Credit**

G51. Did you access rural bank credit during the 2017/2018 farming season? 1=Yes [ ]

0= No [ ]

G52. If Yes, did you get the amount you applied for? 1=Yes, I got the full amount [ ] 2=No, I got less than the amount I applied for [ ] 0=No, I did not get anything at all [ ]

*If the answer to question G52 is either option (1) or (2), then please answer question G53. Otherwise skip to question G54.*

G53.What did you use the credit for? 1=To pay school fees [ ] 2=For food consumption purposes [ ] 3=To purchase cassava farming inputs [ ] 4=To pay rent 5=Other (specify)………………..

G56. Do you have a savings account with the Rural Bank? 1=Yes [ ] 0=No [ ]

G57. Is the credit from the rural bank enough to support your cassava production? 1=Yes [ ] 0=No [ ]

G58. Do you have any other sources of funds to support your cassava production? 1=Yes [ ] 0=No [ ]

If YES, which sources and how much did you get during the 2017/2018 production year?

| Source | Amount (GHS) |
| --- | --- |
|  |  |

**Thank you for your time**
